# Risk control of heavy metal in waste incinerator ash by available solidification scenarios in cement production based on waste flow analysis

**DOI:** 10.1038/s41598-024-56551-y

**Published:** 2024-03-15

**Authors:** Behzad Valizadeh, Mohammad Ali Abdoli, Sina Dobaradaran, Rouhalla Mahmoudkhani, Yousef Abdossalami Asl

**Affiliations:** 1 Department of Environmental Engineering, Faculty of Environment, University of Tehran, Tehran, Iran; 2 Systems Environmental Health and Energy Research Center, The Persian Gulf Biomedical Sciences Research Institute, Bushehr University of Medical Sciences, Bushehr, Iran; 3 Department of Environmental Health Engineering, Faculty of Health and Nutrition, Bushehr University of Medical Sciences, Bushehr, Iran; 4 Department of Environmental Engineering, Science and Research Branch, Islamic Azad University, Tehran, Iran; 5 Reference Laboratory, Health Deputy, Iran University of Medical Sciences, Tehran, Iran

**Keywords:** Municipal solid waste, Incinerator, Solidification, Heavy metals, Environmental sciences, Chemistry

## Abstract

Incineration is a common method in municipal solid waste management, which has several advantages such as reducing the volume of waste, but with concerns about exhaust gas and ash management. In this study, heavy metals in bottom ash, secondary furnace ash and fly ash of two waste incinerators in Tehran and Nowshahr were analyzed and its control in cement production was investigated. For this purpose, twelve monthly samples of three types of incinerator ash were analyzed. By combining the studied ashes in the raw materials, the quantity of metals in the cement was analyzed. Finally, by investigating four scenarios based on quantitative variations in the routes of municipal solid waste, ash quantity and the related risk caused by its heavy metals were studied. The results showed that the concentration of heavy metals in the three ash samples of the studied incinerators was 19,513–23,972 µg/g and the composition of the metals included Hg (less than 0.01%), Pb (2.93%), Cd (0.59%), Cu (21.51%), Zn (58.7%), As (less than 0.01%), Cr (15.88%), and Ni (0.91%). The best quality of produced cement included 20% ash and 10% zeolite, which was the basis of the next calculations. It was estimated that the reduction of the release of metals into the environment includes 37 gr/day in best scenario equal to 10.6 tons/year. Ash solidification can be considered as a complementary solution in waste incinerator management.

## Introduction

The generation of significant volume of solid waste is one of the important consequences of urbanization and globalization, which is a serious environmental concern^1–4^. It is predicted that the global production of solid waste will be 2.2 billion tons by 2025^5^. The collection and safe disposal of solid waste is one of the important challenges in urban management, which has been developed over the past decades by inventing various methods^6^. Approaches such as recycling, recovery, reuse, landfill, and incineration are available in municipal solid waste management. It is very important to select appropriate options from the available approaches based on local social, economic, and technological characteristics with the aim of environmental and health risk control as well as material and energy recovery^7^.

The variety of components in solid waste has caused a significant range of pollutants to be observed in it^8^. For example, can mention heavy metals. Heavy metals are one of the most important pollutants in solid waste, which are present in many wastes such as batteries, electronic waste, and even cigarette butts^2,9, 10^. Due to the presence of these pollutants in recyclable waste, the concentration of heavy metals in municipal solid waste depends on the ratio of separation^1^. In developing countries, due to the lower rate of waste separation, it is likely that the concentration of heavy metals in the waste mass and in by-products such as landfill leachate and incinerator ash is higher^1^. Even considering the importance of pollutant routs, especially air pollution, this condition can affect more people by the release of pollutants from waste incinerators into the air^11,12^. Therefore, the development of efficient management of solid wastes with the aim of increasing the recycling ratio, which leads to the reduction of waste entering landfills and incinerators, can be effective in reducing pollutants caused by solid wastes, including heavy metals^1,2^.

Although landfilling is almost an inevitable measure in waste management, the environmental concerns caused by leachate, as well as the high costs of designing, implementing, and maintaining the landfill site required alternative methods such as composting and incineration to be considered^7^. Incineration is known as a widely used method in municipal solid waste management due to advantages, such as a significant reduction in waste volume and easier operation than other methods such as composting^13^. Reduction of 70% of mass and 90% of volume of municipal solid waste by using incineration was reported in relevant studies^3^. It is also possible to use incineration as an energy generating equipment^3,14^. About 11% of solid waste in the world managed by incinerators^15^.

Despite the significant advantages, a serious concern with incinerators is their byproducts, including pollutant gases, bottom ash, and fly ash^3,13^. Pollutant gases can include toxic and carcinogenic compounds, which is one of the important reasons for the limitations of loading some waste types such as plastic in the incinerator^16^. Also, incineration residues such as bottom ash and fly ash are known as hazardous waste due to the concentration of compounds such as heavy metals^17^. Fly ash consists of particulate matters resulting from burning solid waste and estimated its quantity to be equal to 2% of loaded solid waste, which can be considered as a origin of air pollution^14^. Therefore, air pollution control equipment is one of the requirements in the operation of incinerators, which is usually an important limitation in developing countries. In addition, bottom ash contains a heterogeneous mixture of burned and unburned solid waste components such as metals, ceramics, glass, and partially burned organic matter^18^. The amount of bottom ash is significantly higher compared to fly ash and is reported to be approximately 20% of the solid waste loaded in the incinerator^14^.

Every year, million tons of ash produced in incinerators, which must be properly managed^19^, because bottom ash and fly ash contain a significant concentration of heavy metals, which can be an important pollutant in water, soil, and air^20^. Due to the concerns about the adverse health consequences of heavy metals and other pollutants, in the past decades, various methods have been used to manage incinerators' ash, the most common of which is solidification/stabilization^13^. Also, other methods such as chemical stabilization and vitrification are used for ash management^21^. One of the most common approaches in the solidification method of ash management is the production of building materials, which is considered as a solution for environmental protection and is used in many European countries^20,22^.

In Tehran, 7500 tons of solid waste is generated per day and it is estimated to increase to 9400 tons^23^. Incineration is one of the methods of interest to manage this amount of waste, based on which, in 2014, the first vertical thermal gasification incinerator was established, in which 200 tons of rejected solid waste are loaded, and its energy production capacity is 3 megawatts^32^. Another MSW incinerator with the same capacity and structure, was built in 2020 in Nowshahr city. The aim of this study was to analyze the concentration of heavy metals in the incinerators' ash of Tehran and Nowshahr cities, the ability to use their ash in cement production, and determining the quality characteristics of produced cement. Also, estimation of pollution and pollutant cycle in different ash management scenarios in incineration development plans for municipal solid waste management was another objective of this study.

## Method

### Study area

**Figure 1:**
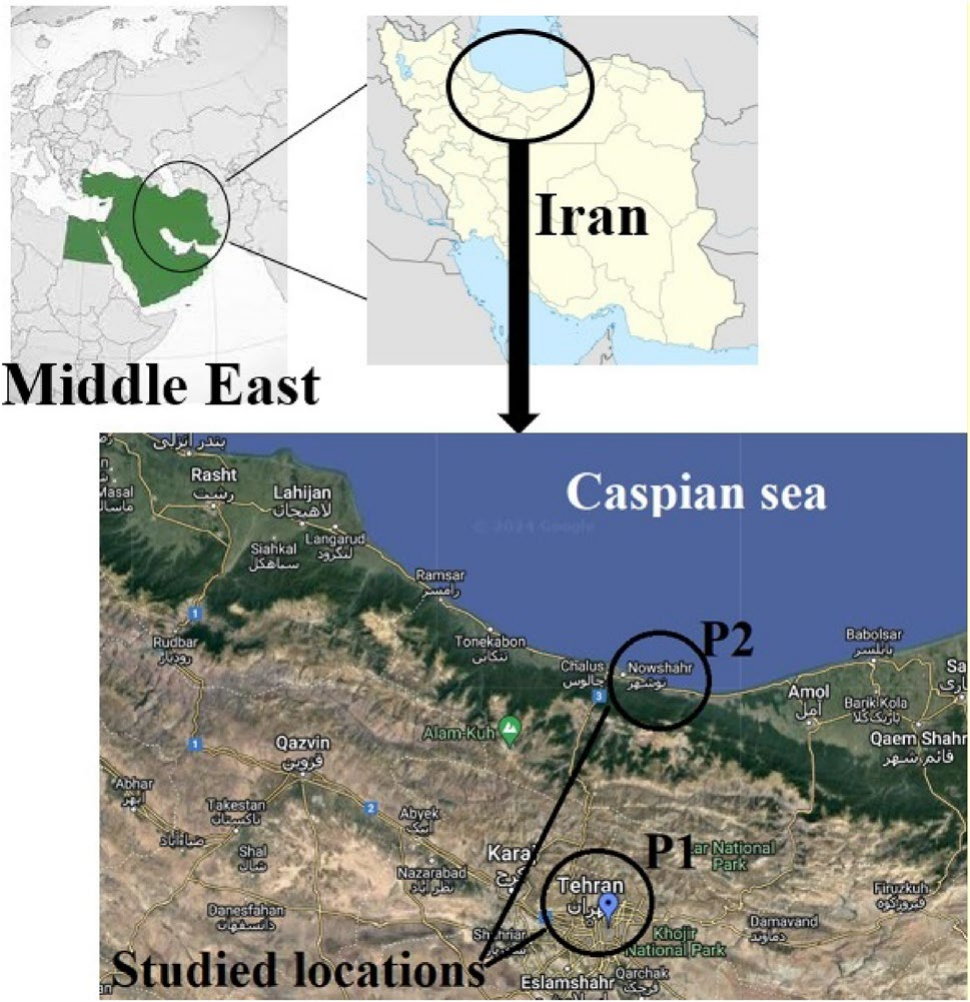
Studied locations: P1 (Tehran), P2 (Nowshahr)-created using Google Earth Pro v7.3.1 [Software].

As shown in Fig. 1, this study was conducted in Tehran and Nowshahr in Iran. Tehran is the capital and has nine million people. The geographical location of the city is in the southern slope of the Alborz Mountain, and includes several climates including mountainous and desert. It has been reported that daily generation of solid waste in Tehran is more than 6200 tons, of which more than 70% is organic waste. Per capita municipal solid waste production in Tehran is 200 g more than the country's average, which is due to the high share of this city in the national GDP. But the city of Nowshahr is located on the northern slope of the Alborz Mountain and its population is 49 thousand people. In the incineration of this city, the municipal solid wastes of Abbas Abad city, with population of thirteen thousand people, is also loaded.

These cities, which are located on the coast of the Caspian sea, have a temperate mountain climate and a high humidity ratio. As shown in Table 1, the per capita generation of municipal solid waste in Nowshahr and Abbas Abad is lower than Tehran, and the composition of municipal solid waste is not the same compared to Tehran. However, solid waste management has the same pattern in both studied locations. Considering the total rate of source separation which is less than 15% in both cities and geographical locations, the main option for waste management in Tehran and Nowshahr is landfilling and incineration respectively.

**Table 1:** Solid waste composition in studied cities^28^.

	Per capita waste generation (g/day)	Composition (%)					
		Organic	Paper	Metals	Plastic	Glass	Other
Tehran	840	74.56	5.04	2.48	6.25	2.03	9.64
Nowshahr	760	77.72	8.43	0.89	7.61	0.91	4.44

### Operation of incinerators

**Figure 2:**
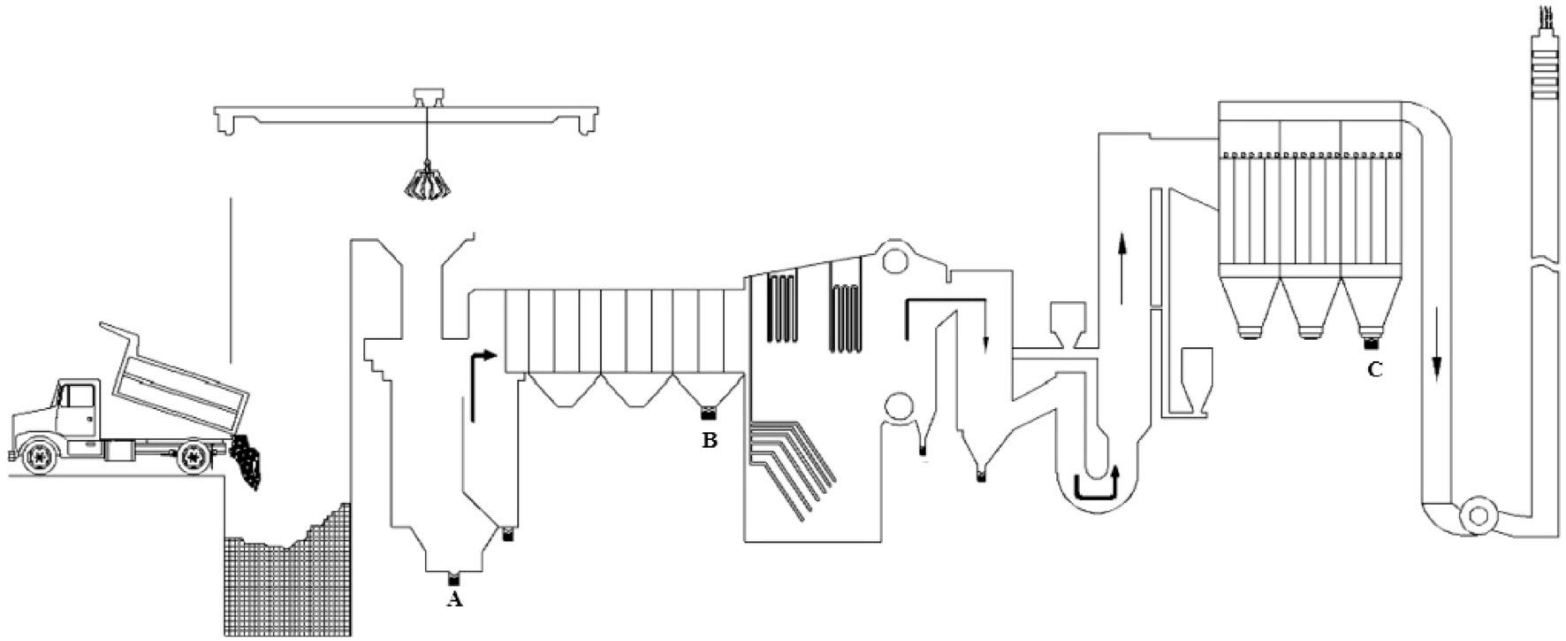
Schematic of the incineration plant and different ash producing location; (**A**) Bottom Ash, (**B**) Secondary Furnace Ash, (**C**) Fly Ash^32^.

Dual chamber incinerators were installed in Tehran and Nowshahr in 2014 and 2020, respectively. The capacity of the studied incinerators was similar. However, the daily waste loading in Tehran incinerator and Nowshahr incinerator was 200 tons and 179 tons, respectively. Thematic parts of incinerators is shown in Fig. 2. The combustion process was carried out in the first chamber and the second chamber at the temperature of 850–1050 °C and 950–1200 °C, respectively. The flue gas retention time is more than 2 s and heated flue gas is passed through a boiler which is equipped with a super heater and economizer. Hydrated lime sorbents and activated carbon are applied to remove acid gases (mainly HCl, HF, and SO_2_) and to capture volatile heavy metals, dioxins, micro-organic pollutants in baghouse filter.

### Sampling and analysis

As shown in Fig. 2, three ash samples, including Bottom ash (BA), Secondary Furnace Ash (SFA), and Fly Ash (FA), were received from the points A, B, and C, respectively. Sampling was done in both Tehran (P1) and Nowshahr (P2) incinerators in four seasons. In each season, nine samples were taken from each of the studied ashes. The weight of each sample was 1 kg. Therefore, in total of four sampling periods, 36 samples were studied. Sample processing included drying and homogenization. First, the samples were dried for 24 h at a 60 °C, then a sieve was used to homogenize the samples and separate the coarse pieces. The analysis of metals was done using the acid digestion method. 0.5 g of the processed sample was digested in the presence of 65% nitric acid and 37% hydrochloric acid with the help of microwave digester^24^. Accordingly, concentration of metals was analyzed by ICP-OES. Arsenic analysis done by a hydride generation technique. Mercury analysis done by the Mercury Analyzer (milestone-DMA-80).

### Cement production and leachate analysis

In a processing step, large and unburned iron pieces were removed from the ash samples using a magnet. For further treatment, crushing and screening of the samples were done to prepare a homogeneous composition in terms of size at room temperature. Cement samples were made in 50 mm^3^. In the production of cement, the ratio of 70:20:10 including BA, SFA, FA was used. The ratio of ash in the raw material of concrete production included 25%, 35%, and 50%. Zeolite was used in the ratio of 10% and 20% to investigate the improvement of the compressive strength of concrete. The strength test was performed according to ASTM C39.

Then, the concrete samples were crushed in dimensions of about 9.5 mm. The extraction solution was prepared according to the EPA 1311 method and was added to the samples with a liquid-to-solid ratio of 20. The samples were shaken in the extraction solution for 18 h at 30 rpm. Then, the supernatant of the samples was extracted and digested by a microwave digester. The desired metals were measured by ICPOES and mercury analyzer after the digestion process.

### Scenarios

In this study, four scenarios for solid waste management using incinerator were evaluated. As shown in Table 2, in the first scenario, it was assumed to increase the capacity of Tehran incinerator up to 2000 tons. This scenario was defined based on the generation of 4600 tons/day of organic waste in Tehran. The second scenario included the reduction of organic waste generation by improving the food waste management hierarchy up to 1000 tons per day and increasing the capacity of the waste incinerator up to 1000 tons. The third scenario included increasing the capacity of compost up to 1500 tons per day to reduce the waste loading on the incinerator and increasing the capacity of the incinerator up to 500 tons. The fourth scenario as a single-option method included increasing the capacity of the incinerator up to 2500 tons per day.

**Table 2:** Fate of solid waste in studied scenarios.

Scenarios	Management options (%)			
	3R	Landfill	Compost	Incineration
S1	15	34.89	17.57	32.54
S2	17.92	41.64	20.99	19.45
S3	17.92	43.22	29.15	9.71
S4	17.92	12.50	20.99	48.59

## Results and discussion

**Figure 3:**
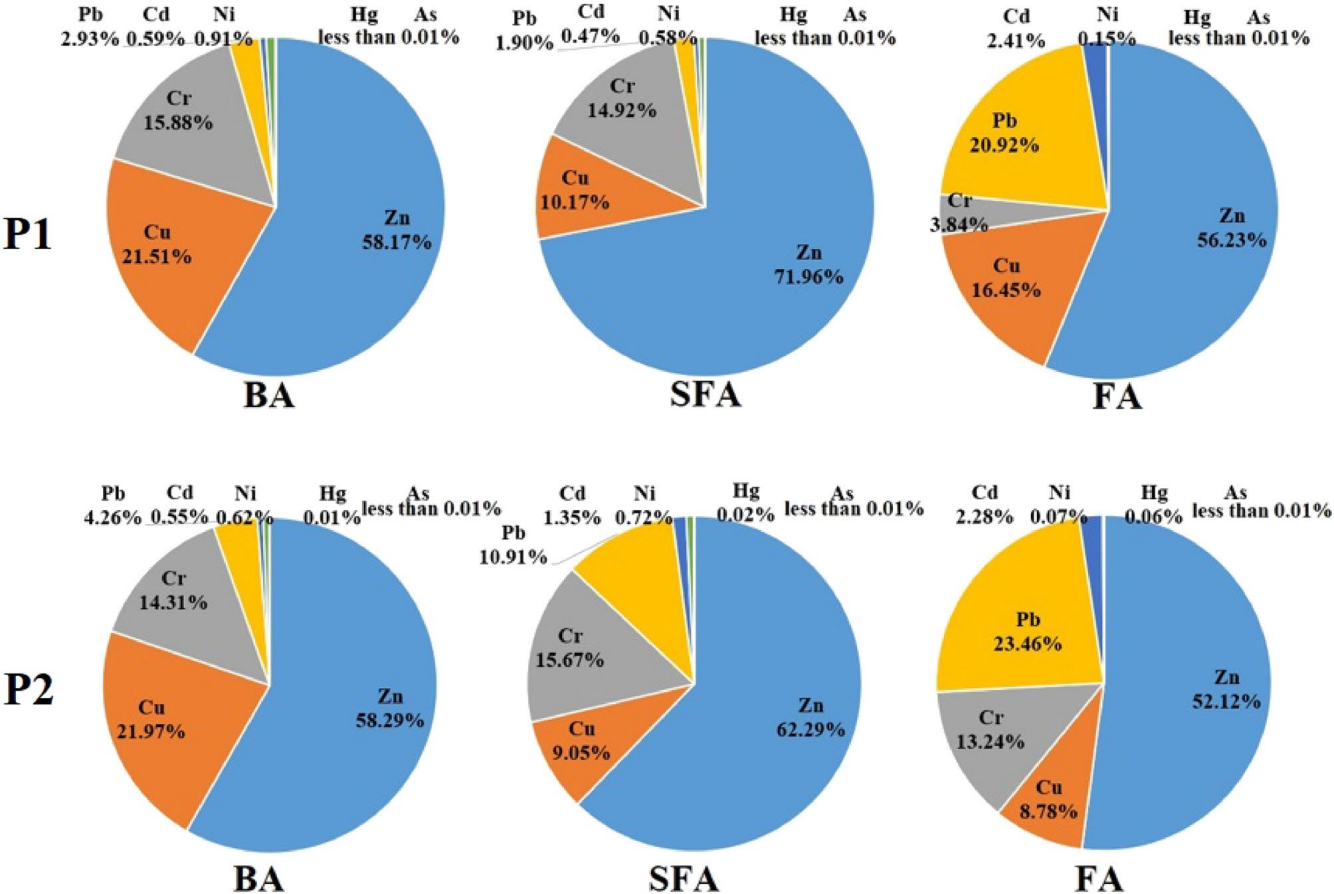
The proportion of heavy metals in the studied ashes.

The results showed that due to the incineration of 379 tons of solid waste in the cities of Tehran and Nowshahr, 83.4 tons/day of ash are produced as a byproduct. The capacity of Tehran incinerator was equal to 3.22% of municipal solid waste and 4.6% of organic waste. The use of incinerator in Nowshahr is considered as a solution for solid waste management due to the limitations of the landfill in this area, such as low depth of groundwater, high rainfall, and lack of available land due to population density. However, because the climatic and geographical conditions of Tehran do not have these restrictions, landfill is the main option for disposing of municipal solid waste. In this situation, the main question is *whether the incinerator can provide a safe option for municipal solid waste management in the two studied areas?* Although the main concern in using the incinerator is to control the exhaust gas with many pollutants including dioxin, furan, SOx, and NOx^16^, but ash can also be considered as an environmental threat^25^. One of the most important pollutants in ash is heavy metals, which, if not properly managed, can be transferred to soil and water resources and even enter the food chain^26^. The results of heavy metals concentration analysis in the studied ash samples are shown in Table 3. Zinc had the highest concentration among the studied metals, and mercury and arsenic had the lowest concentration in the studied ashes. However, the ratio of metals was different in the analyzed samples. As shown in Fig. 3, the share of zinc in the composition of investigated metals was 52% to 72% in different samples.

**Table 3:** Heavy metals concentration in studied ash samples.

	Metals (µg/g)							
	Zn	Cu	Cr	Pb	Cd	Ni	Hg	As
P1								
BA								
Spring	1696.13	1097.90	430.48	157.73	20.12	38.90	0.051	0.099
Summer	2028.65	537.38	411.05	74.32	12.32	15.14	0.01	0.081
Autumn	1950.21	475.28	671.26	1.36	26.47	21.72	0.021	0.142
Winter	1537.11	556.56	455.50	130.09	14.07	37.36	0.013	0.293
SFA								
Spring	2366.87	424.80	488.56	64.39	16.3	22.98	0.001	0.047
Summer	3068.62	370.31	627.53	76.60	18	23.10	0.004	0.341
Autumn	2566.08	525.23	540.82	66.50	18.74	17.39	0.003	0.014
Winter	2773.41	202.35	576.64	77.50	16.94	23.84	0.004	0.065
FA								
Spring	7380.12	2551.12	466.78	3197.23	397.7	18.86	0.31	0.201
Summer	8004.99	2025.04	336.53	3029.88	345.04	14.80	0.121	0.06
Autumn	5374.87	1742.83	564.72	1790.57	194.71	23.96	0.523	0.481
Winter	7737.5	2019.82	576.17	2586.06	285.49	16.73	0.323	0.012
P2								
BA								
Spring	1105.17	504.38	371.69	142.12	14.14	18.99	0.011	0
Summer	2668.85	645.48	315.71	73.29	12.75	10.78	0.023	0.006
Autumn	991.75	429.28	373.74	124.34	13	18.10	1.031	0.23
Winter	1008.04	596.77	356.39	82.27	14.29	13.42	0.044	0.08
SFA								
Spring	3342.46	346.47	371.65	1102.60	124.27	38.14	2.081	0.124
Summer	1473.03	325.19	526.85	81.54	13.24	15.87	0.014	0
Autumn	1404	252.55	517.68	72.46	15.34	12.86	0.116	0.198
Winter	1408.21	183.46	502.62	78.90	12.56	20.75	0.652	0.077
FA								
Spring	10,960.03	1476.27	301.80	5702.26	626.02	8.62	15.961	0.253
Summer	12,770.4	1681.49	8829.45	5787.78	484.49	14.26	5.302	0.177
Autumn	8312.56	1645.11	245.24	3254.94	290.03	13.84	19.57	0.105
Winter	6387.37	1670.37	390.21	2555.06	277.5	11.26	1.453	0.185

The total concentration of studied metals in BA,P1 was 3099.4 µg/g, while the total concentration of metals studied in BA,P2 was 2476.5 µg/g. Also, the highest concentration was seen in FA samples, which were 12,670.9 µg/g and 18,434.8 µg/g in P1 and P2, respectively. Also, the information in Table 3 shows that the concentration of studied heavy metals in spring and summer samples was higher than autumn and winter samples. Although the concentration of studied heavy metals in BA and SFA was higher in Tehran incinerator, but the concentration of studied heavy metals in FA was higher in Nowshahr incinerator. Therefore, the concentration of metals in incinerator ash had temporal and spatial variations. As shown in Fig. 2, the fly ash contains the highest concentration of heavy metals due to their evaporation during combustion and subsequent absorption onto the surface of fly ash particles^27^. The fly ash in P1 had concentration of various metals descending order as follows: Pb > Hg > Cd > Cu > Zn > As > Cr > Ni. While, when considering P2, there is a negligible shift in proportions: Hg > Pb > Cd > Zn > Cu > Cr > As > Ni. Clearly evident is the fact that within both plants, a considerable quantity of lead, mercury, cadmium, copper, and zinc has culminated in fly ash. This phenomenon can be ascribed to various factors including high steam pressure and low melting temperature^14^. Additionally, it is worth noting that the highest accumulation of arsenic, nickel, and chromium takes place specifically within BA and SFA.

The different composition of municipal solid wastes in different seasons and in different cities can be effective in the observed temporal and spatial variations. Although the composition of municipal solid waste in Iran has a similar pattern^28^, but there may be slight differencess in different cities. The difference in the component of loaded solid waste in the incinerator such as plastic, paper and cardboard, and food waste causes the difference in the concentration of metals in the ash. For example, a higher proportion of paper and leather in the waste leads to increased levels of lead^29^. Also, plastic waste have an impact on concentration of chromium and cadmium in ash^29,30^.

In addition, some components of municipal solid waste have a diverse range of heavy metals, and changing their ratio in the loaded waste in the incinerator has an effect on the concentration of ash metals. For example, electronic waste and cigarette butts have various concentrations of heavy metals^2,31^. The management of these wastes, which are classified as hazardous wastes, is effective in the quantity of their loading in the incinerator. The low rate of source separation in Iran, such as cigarette butts, battery wastes, and electronic waste causes an increase in the amount of waste containing heavy metals in the mass loaded in the incinerator^31^. Table 2 provides a clear depiction of the metallic content within the samples, with zinc metal prevailing as the highest recorded amount. This can potentially be attributed to the significant presence of textile and food waste^29,32^. On the other hand, the origin of metals such as arsenic and mercury are wastes such as pesticides, chemotherapy drugs, thermometers, fluorescent lamps, and batteries^33,34^, which their proportion in the composition of municipal solid waste in Iran is much lower than that of food waste^1^. Therefore, the lower concentration of mercury and arsenic in the studied ash samples was due to the composition of municipal solid waste loaded in incinerators.

One of the primary causes for the disparity in metal concentrations, specifically copper and zinc, between BA, SFA, and FA can be attributed to their mass^35^. This mass is entirely transferred to both exhaust gases and fly ash^36^. Temperature plays a crucial role in the transfer of lead to both gas phase and fly ash; as temperature increases, so does the rate of lead transfer^36^. However, metals like nickel, arsenic, and chromium tend to accumulate more in bottom ash. These findings align with other studies conducted within this field^29^. Nickel and chromium are considered non-volatile metals with relatively high masses; as a result, their transfer into the ash is less pronounced^29,35, 36^. A noteworthy portion of total arsenic content remains within the fly ash due to its reaction with calcium oxide or iron oxide. This reaction leads to the formation of non-volatile compounds^37^.

**Figure 4:**
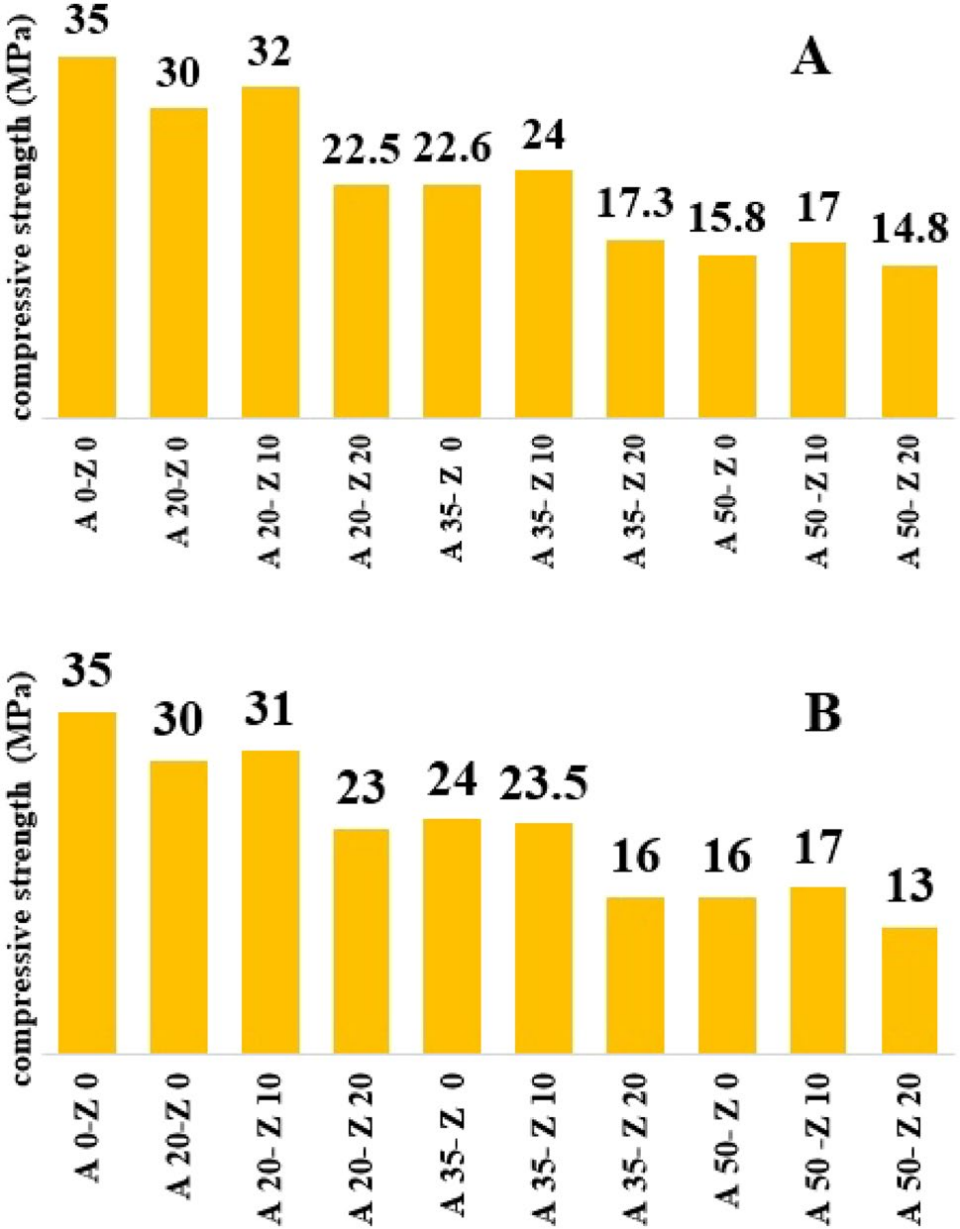
The amount of compressive strength (**A**) plant 1, (**B**) plant 2 (A used for mixed ash percentage and Z used for natural zeolite percentage).

The appropriate manner in which to handle the remaining residue from the incinerator is through recycling and reuse. Using ash in the production of concrete serves both to achieve this objective and effectively regulate metal leakage^27^. Various factors must be assessed to determine the level of strength. To evaluate the durability of concrete incorporating residual ash and natural zeolite, one such factor termed compressive strength was employed in this study, with outcomes presented in Fig. 4. Within general construction parameters, a value ranging between 15 and 30 MPa was deemed acceptable^38^. Notably, the sample containing a combination of 20% mixed ash and 10% zeolite demonstrated the highest compressive strength at 32 MPa. It is evident that as the proportion of ash increases, so does the weakness within its structural integrity; however, an inclusion rate of 10% zeolite enhances overall strength when compared to employing natural zeolite at a rate of 20%. The incorporation of natural zeolite as an additive substantively enhances the pozzolanic reaction in concrete, resulting in a superior sustenance of compressive strength over time. Besides this notable benefit, there exist several other advantages associated with its application. These include a reduction in speculation, suppression of the alkali-aggregate reaction, and enhancement of physical properties. Significantly, empirical evidence from previous studies reinforces these findings by demonstrating decreased compressive strength under comparable conditions^39^.

The leaching test stands as the most contentious matter within waste management. This evaluative measure serves to assess the potential environmental impact by determining the release of metals from incinerator residual ash. The prerequisites for this procedure entail creating a uniform sample comprised of collected BA, SFA, and FA. These samples, representing an annual average index, are obtained from each plant over various seasons. Table 4 shows the measured metal concentrations resulting from the mentioned test, in comparison to standard values established by USEPA. Furthermore, a benchmark for similar leakage testing processes hails not only from the EPA but also from selected nations such as China, Taiwan, and Singapore^32^.

**Table 4:** The heavy metal leaching concentration in separate samples of BA, SFA, and FA for both plants.

	Cr	Cu	Ni	Pb	Zn	Cd	Hg	As
P1								
BA	480	3200	23	575	598	1	0.009	0.007
SFA	776	2848	8	562	391	12	0.005	0.008
FA	623	3482	90	2003	1821	424	0.02	0.057
P2								
BA	400	2763	12	326	558	0.1	0.012	0.005
SFA	530	1256	11	999	264	32	0.008	0.006
FA	610	3164	35	2785	2341	568	0.12	0.01
US EPA								
Standard	5	100	–	5	–	1	0.2	5
Chinese standard	15	100	5	5	100	1		5
Taiwan standard	5	15	–	5	–	1	0.025	5
Singapore standard	5	100	5	5	100	1		5

In general, the leaching of metals is influenced by various factors, including the geological and hydrological conditions. pH levels, liquid-to-solid ratio, particle sizes, weather conditions, and aging also contribute to the amount of metal leakage. Among these factors, pH and liquid-to-solid ratio are deemed more significant^40^, as indicated in the provided Table 4. Regarding arsenic leakage, all samples exhibit quantities below the permitted limit in four references. However, concerning mercury leakage, only the fly ash sample from P2 surpasses the permissible threshold. The quantity of leaked cadmium, copper, zinc, nickel, and lead significantly exceeds the acceptable limit in all specimens. The presence of food waste and plastic compounds in the input waste accelerates metal washout rates while facilitating their movement–particularly Pb, Cr, Cd^32,40^. Moreover, organic compounds may be responsible for elevated copper leakages^41^. Additionally, the amount of leaked metals is contingent on their initial concentration within ash materials^32^.

The concoction crafted for incorporation into concrete, which consists of BA, SFA, and FA in proportions of 70%, 10%, and 20% respectively, underwent a test to assess its leakage both before and after being utilized in the concrete. This examination was conducted under identical circumstances for two plants, with the results presented in Table 5 for comparison. As discernible from the findings, there has been a noticeable reduction in the amount of metal leakage subsequent to its implementation in the concrete. Moreover, this reduced quantity falls within the permissible range established by USEPA's guidelines. Additionally, it adheres to the set standards imposed by China, Singapore, and Taiwan as well. The metals involved in the process of concrete production are rendered immobile during hydration—they become fixed within the structure due to this chemical reaction. The extent of their potential leakage is contingent upon compounds containing metals solubility. Furthermore, alkalinity serves as an element that diminishes said solubility. Consequently, incorporating natural zeolite further contributes to minimizing metal leakage owing to its impact on alkalinity^18,39, 42^.

**Table 5:** The heavy metal leaching concentration of mixed ashes (mg/l).

	Mixed ash-P1		Mixed ash-P2	
	Before concrete production	After concrete production	Before concrete production	After concrete production
Cr	538.2	4.8	455	3.7
Cu	3221.2	125.6	2692.5	82.4
Ni	34.9	3.4	16.5	3.2
Pb	859.3	2.4	885.1	2.2
Zn	821.9	97.8	885.2	91.9
Cd	86.7	0.76	116.87	0.84
Hg	0.0108	0.00002	0.0332	0.00061
As	0.0171	0.00034	0.0061	0.0002

**Figure 5:**
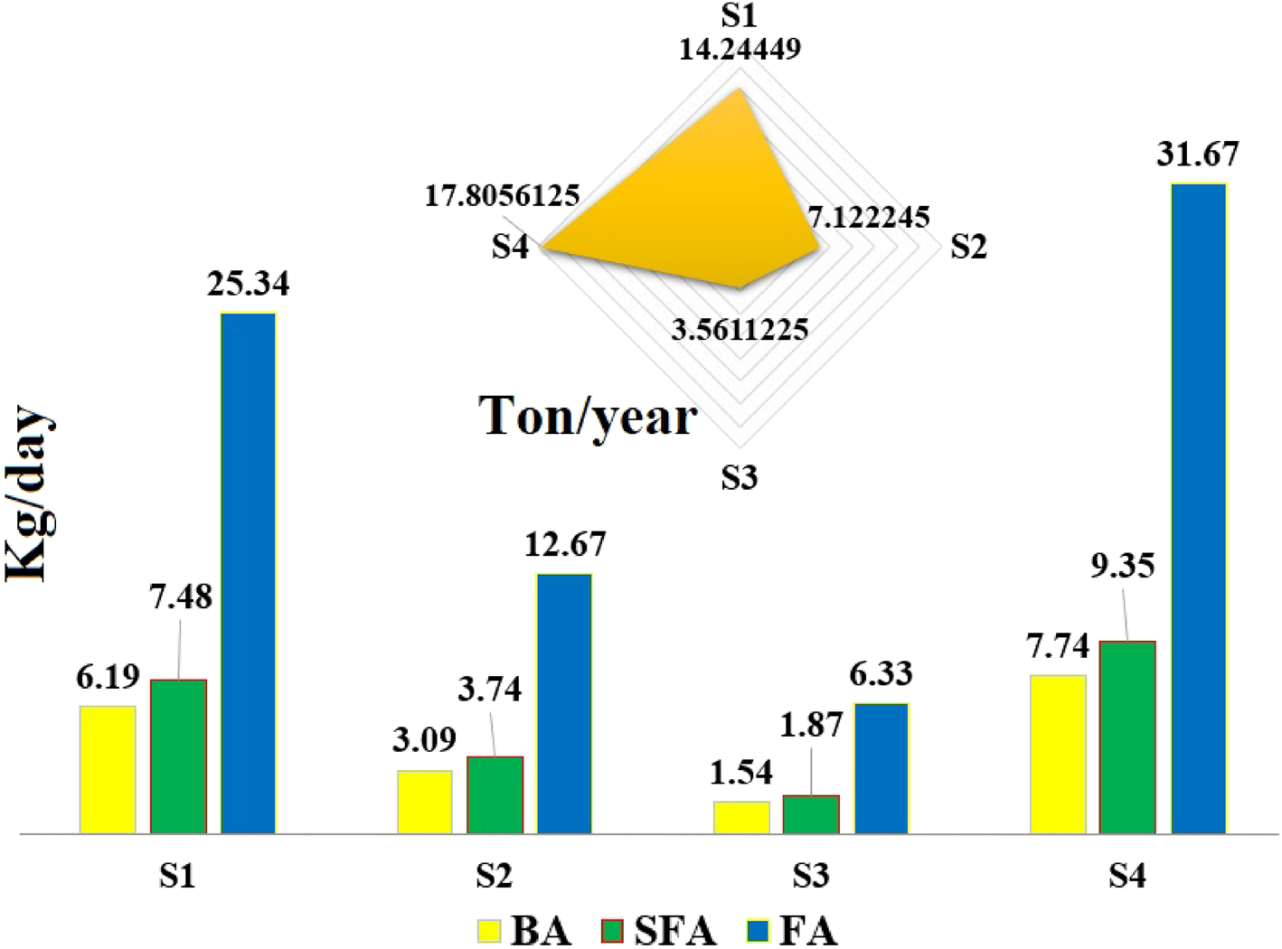
The estimated quantity of heavy metals caused by the incineration of solid waste in the studied scenarios.

The results of estimating the quantity of heavy metals in the studied scenarios are shown in Fig. 5. The results showed that the lowest quantity of heavy metals was estimated in the third scenario and the fourth scenario had the highest quantity of heavy metals. Therefore, the tendency to develop incineration will be associated with a significant increase in the risk of heavy metals in ash. For this reason, predicting the environmental consequences in choosing solid waste management development options is a necessity, which is done by technical–economic analysis of different scenarios^1^. For example, the development of methods to reduce waste production through the replacement of alkaline batteries with rechargeable batteries had the best effect in the environmental and economic consequences of different battery waste options in Iran^2^. In this study, the positive effect of reducing waste production and increasing compost capacity in the second and third scenarios reduced the environmental risk caused by heavy metals in solid waste management. The result showed that the risk of heavy metals emission into the environment in the second scenario was 50% lower than the first scenario, while the use of the third scenario as solid waste management option reduces the environmental risk of heavy metals by 80% compared to the fourth scenario. Considering that one of the important factors in recycling solid waste or trapping pollution in recycled products such as concrete is its capacity^43^, and the results of this study, which showed that 20% of ash and 10% zeolite ratio is optimal for producing concrete, it is necessary to estimate the quantity of concrete to accept ash.

The amount of concrete required for encapsulating ash in the third scenario is 200,750 tons/year, while 1,003,750 tons of concrete must be produced annually to manage incinerator ash by producing concrete in the fourth scenario. Therefore, in the best ratio for the production of quality concrete, the heavy metal control factor is estimated to be 0.0000177, which indicates the trapping of 17.7 g of heavy metal in each ton of concrete. The limitations of landfill method for incineration ash due to environmental concerns is a challenge for using incinerators. On the other hand, due to the impact of social factors on waste production and also its management solutions, such as the desire to use recycled products, the use of ash in the preparation of concrete for residential, commercial, and office building materials is limited. Also, due to technical concerns about the quality of concrete with ash ratio in raw materials, it limits the application of this method in the production of concrete for roads and bridges. Therefore, according to the mentioned the heavy metal control factor and the amount of ash production in the studied scenarios, the unlimited development of incineration without considering the consequences of ash can challenge the management of municipal solid waste.

This study had strengths and limitations. The analysis of all types of incinerator ashes was one of the strengths of this study. Considering possible variations in the quality of municipal solid wastes due to seasonal changes, and monthly sampling was another strength of this study. Another strength of this study was investigating the fate of the pollutant in the stages of ash management for cement production. However, the lack of measurement of other pollutants such as microplastics was a limitation of this study. Although pollutant monitoring in ash recycling scenarios was complete, the lack of pollutant monitoring in other ash management options such as landfill was a limitation in this study that can be considered in future studies.

## Conclusion

The concentration of heavy metals in the ash of Tehran and Nowshahr incinerators and the possibility of its control in concrete production were studied. The results showed that the total concentration of the eight studied heavy metals in the ashes of Tehran and Nowshahr incinerators was 19,513 and 23,972 µg/g, respectively. The proportion of metals in the studied ashes was not the same, where zinc had the highest ratio and mercury and arsenic had the lowest ratio in the concentration of metals. The ratio of 20% of ash and 10% zeolite in the raw materials of concrete led to the production of concrete with the best mechanical strength. However, as the proportion of the remaining ash mixture increased in all samples, a decrease in strength became evident. Nevertheless, barring the sample containing 50% mixed ash and 20% zeolite; all other samples fell within an acceptable range of 15 to 30 MPa. The encapsulation coefficient of heavy metals in concrete was 1.77 g/ton. Based on this, the annual need for recycled concrete production for Tehran incinerator ash management in the lowest and highest scenario was estimated by 200,750 tons and 100,3750 tons, respectively. Therefore, although the development of incineration in the management of municipal solid waste can seem useful, it is necessary to pay attention to the environmental consequences of ash and the technical–economic limitations of its management.

## Data Availability

The datasets generated and analyzed during the current study available from the corresponding author on reasonable request.
